# Using PINS pulses to saturate inflow effects on fMRI data at 3 and 7 T

**DOI:** 10.1002/mrm.30584

**Published:** 2025-05-20

**Authors:** Shota Hodono, Chia‐Yin Wu, Jin Jin, Jonathan R. Polimeni, Martijn A. Cloos

**Affiliations:** ^1^ Donders Centre for Cognitive Neuroimaging Radboud University Nijmegen Netherlands; ^2^ Centre for Advanced Imaging The University of Queensland St. Lucia Queensland Australia; ^3^ Imaging Centre for Excellence University of Glasgow Glasgow UK; ^4^ Siemens Healthineers Pty Ltd Brisbane Queensland Australia; ^5^ Athinoula A. Martinos Center for Biomedical Imaging, Department of Radiology, Harvard Medical School Massachusetts General Hospital Charlestown Massachusetts USA; ^6^ Harvard–MIT Program in Health Sciences and Technology MIT Cambridge Massachusetts USA

**Keywords:** extravascular BOLD, inflow effect, intravascular BOLD, power independent of number of slices (PINS) pulse, SE‐BOLD

## Abstract

**Purpose:**

To suppress inflow effects by saturating the magnetization within slice gaps.

**Methods:**

Power independent of number of slices (PINS) pulses was designed to saturate the magnetization in all slice gaps at once. The PINS saturation module was played before every excitation. The saturation and excitation profiles were validated in simulation and phantom experiments. To demonstrate the efficacy of the method to suppress inflow, experiments were performed using a flow phantom. As an example use‐case, fMRI experiments with and without PINS inflow saturation were performed at 3 T and 7 T.

**Results:**

Simulations and phantom experiments showed that the PINS saturation module successfully saturated the magnetization in the slice gaps without degrading the slice profile of the imaging slices. Flow phantom experiments showed that the PINS saturation module suppresses through‐plane inflow better than no‐gap acquisitions. In vivo fMRI experiments demonstrated that the PINS saturation module can be used to modulate the spin‐echo BOLD signal. At 3 T application of PINS pulses to saturate the magnetization in the slice gaps resulted in approximately 25% fewer activated voxels (PINS‐ON vs. PINS‐OFF). Interestingly, at 7 T the activation patterns remained more similar and only approximately 10% fewer activated voxels were detected. The observed difference between 3 and 7 T may be linked to the relative shortening of the blood T_2_.

**Conclusion:**

Using PINS pulses, inflow effects from slice gaps were effectively and efficiently saturated. The proposed PINS saturation module can be used to further study the contribution of inflow effects in fMRI data.

## 1 INTRODUCTION

Inflow effects in MRI can be a desirable source of contrast,^1^, ^2^, ^3^, ^4^, ^5^ or an unwanted source of artifacts and bias.^6^, ^7^, ^8^, ^9^, ^10^ Inflow effects are particularly strong when the steady‐state magnetization in the imaging slice is much smaller than at equilibrium. When fluids, such as blood or CSF, enter the imaging slice they carry along fresh magnetization. When the rate of flow is large, relative to the ratio between the slice thickness and TR, bright‐fluid signals emerge.

In fMRI, neural activity is inferred from hemodynamic changes induced through neurovascular coupling.^11^ Most fMRI experiments are based on the BOLD contrast,^12^ which can be measured using gradient recalled echo (GRE) and spin‐echo (SE) sequences. However, both GRE‐ and SE‐BOLD signals are also shaped by local changes in cerebral blood volume (CBV) and cerebral blood flow (CBF).^13^ Inflow effects can alter the relative weighting of these contributions to the overall fMRI signal.

It is thought that the hemodynamic response is initiated by CBF changes in the capillary bed of the parenchyma, providing the highest spatiotemporal specificity.^14^, ^15^, ^16^, ^17^, ^18^ Therefore, the relative contribution of CBF, CBV, and BOLD effects can change not only the amplitude of fMRI signal, but also the specificity. Left unchecked, the inflow effects might skew the interpretation of fMRI data.^6^, ^7^, ^8^, ^9^, ^10^

Previously the role of inflow effects in fMRI has been studied using T_1_ weighting,^7^, ^19^ small diffusion‐weighting gradients,^20^ multi‐echo acquisitions,^10^ or saturation bands. Dedicated saturation bands can be placed outside the imaging volume to saturate the magnetization of inflowing blood^21^; the effectiveness of this approach depends on the number of imaging slices. When a large number of slices are imaged, the longitudinal magnetization of the blood can recover before leaving the imaging volume. Inversion recovery based blood saturation methods can be applied globally, therefore allowing effective saturation in a large FOV. Vascular‐space‐occupancy fMRI,^22^ for example, leverages an inversion recovery strategy to obtain a CBV weighted functional signal. However, this approach also changes the signal intensities of the brain tissue itself. To study the effect of inflow in conventional BOLD fMRI strategies, saturation strategies are needed that do not alter the tissue contrast.

We identified an opportunity to saturate inflowing blood taking advantage of the gaps between imaging slices found in some fMRI protocols. To minimize parasitic saturation effects because of overlapping slice profiles, slices are commonly acquired in an interleaved fashion.^22^ However, interleaved acquisitions cannot fully eliminate slice cross‐talk.^22^ In particular, when SE sequences are used, the refocusing pulse may have a slice profile that is wider than that of the excitation pulse, potentially exacerbating cross‐talk effects. To further mitigate the cross‐talk between slices, a slice gap can be introduced.^23^, ^24^ However, the steady‐state longitudinal magnetization in these gaps can be substantially larger than within the slices themselves. Consequently, when fluids such as blood and CSF move from these gaps into an image slice, their large magnetization can cause a pronounced change in signal.

In this study, we demonstrate the use of power independent of number of slices (PINS) pulses^25^ to efficiently saturate the magnetizations in all slice gaps. The PINS pulse is an undersampled slice selective sinc pulse that excites an infinite stack of slices with a pre‐defined periodicity. Therefore, the PINS saturation pulse can be used to saturate the magnetizations in all slice gaps at once. Moreover, the PINS pulse can also saturate blood far beyond the imaging slice, which may help suppress very fast flowing blood before reaching the imaging volume.

To demonstrate the proposed method, we implanted the PINS saturation pulses in a SE EPI sequence, because SE‐EPI protocols frequently have slice gaps. The PINS module was carefully designed to saturate magnetization in slice gaps with minimum influence on the imaging slices. The method was validated in simulation and flow‐phantom experiments. In vivo experiments were performed at 3 T and 7 T to highlight how inflow and intravascular contributions alter the fMRI signal.

## 2 METHODS

### 2.1 Sequence and PINS pulse design

**FIGURE 1:**
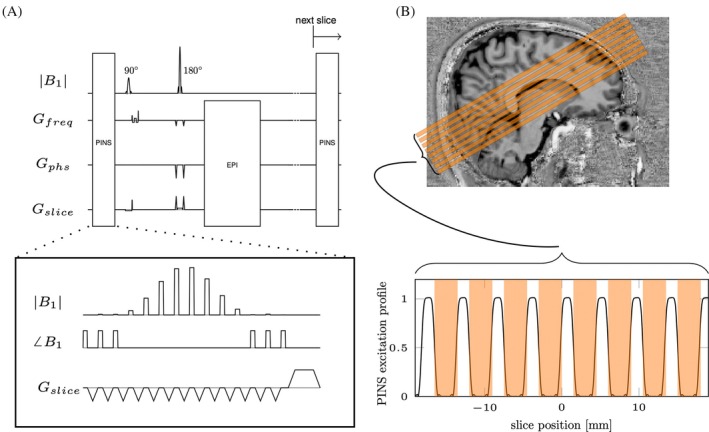
(A) Sequence diagram of power independent of number of slices (PINS)‐spin‐echo (SE)‐EPI sequence. PINS pulse module is played before every excitation pulse. (B) Slice positioning on the visual cortex and the simulated PINS saturation profile. Orange areas represent imaging slices.

The proposed PINS saturation module was played before each excitation in a SE‐EPI sequence (Figure 1A). Therefore, all slice gaps experience the saturation pulse *N*/*M* times per volume TR, where *N* is the number of slices and *M* is the multiband factor.

In this study, the SE‐EPI experiments used a 3‐mm slice thickness with a 50% slice gap. The PINS pulse was designed to saturate the longitudinal magnetization in all the gaps (Figure 1B). The time‐bandwidth product (TBP) for a sinc RF pulse with a Dirac comb function is given by

$$\mathrm{TBP}=\frac{d}{D}\times N_{\mathrm{sub}},$$

where *d*, *D*, and *N*_sub_ are saturation slice thickness (slice gap), periodicity, and number of subpeaks of PINS pulse.^26^ The gradient moment played between each subpulse is

$$A_{\text{blip}}=\frac{2\pi }{\gamma }\times \frac{1}{D},$$

where *γ* is the gyromagnetic ratio.^26^ Many different PINS configurations are possible. Here, we settled at a heuristically optimized PINS pulse configuration (*N*_*sub*_ = 14, *d* = 1.0 mm, *D* = 4.5 mm, flip angle = 90°). In this case, the 1.0 mm of saturation thickness was used considering the non‐ideal saturation profile. Higher TBP allow sharper saturation profiles in space, but are constrained by specific absorption rate and gradient performance limitations.

Bloch simulations were performed to evaluate the available longitudinal magnetization in the slice gaps with and without the PINS module. In addition, dB_0_ and B_1_ influences on the saturation profile were also investigated. The software source code used to design and simulate the PINS pulse can be found at github (https://github.com/s‐hodono/PINSpulse_sim), allowing exploration of other configurations.

### 2.2 Phantom measurements

Phantom experiments were performed to empirically evaluate the efficacy of the PINS “saturation” pulse profile and to evaluate efficacy of inflow saturation. All phantom experiments were performed at 3 T (Prisma, Siemens Healthineers) using the 64‐channel head–neck coil. The SE‐EPI protocol parameter values were: slice thickness = 3 mm, 0 or 50% gap, volume TR = 2 s, TE =75 ms, matrix size = 76 × 76, and 3‐mm isotropic resolution.

In a saturation pulse profile experiment, a 16‐cm diameter spherical phantom (1.25 g/L NaCl) was used to measure the saturation profile produced by the PINS module. Only one imaging slice was used with above sequence parameters. The single imaging slice was shifted ±6 mm (in steps of 0.05 mm) from isocenter without shifting the PINS pulse. The acquired MRI signal amplitude was then plotted as a function of the shift distance.

To evaluate efficacy of inflow saturation, a home‐built cylindrical flow phantom^27^ was scanned. The flow was provided by a water tank and pump control system placed in a console room, regulated by an Arduino. The flow rate was approximately 1.5 cm/s. Three scans (8 imaging slices) were compared: without slice gap, with a 50% slice gap, and with a 50% slice gap + PINS‐saturation.

### 2.3 Evaluation of inflow contribution to fMRI signal

Two healthy volunteers (2 females, 32.5 ± 4.5 years old) with normal/corrected vision, having provided prior, written informed consent, ware scanned at 3 T and 7 T. The study was approved by the local human research ethics committee in accordance with national guidelines.

At 3 T (Prisma, Siemens Healthineers) the 64‐channel head–neck coil was used in combination with the circularly polarized body coil for transmission. At 7 T (Magnetom 7 T+, Siemens Healthineers) the single transmit (circularly polarized) 32‐channel head coil (Nova Medical) was used. First, an anatomical image with a 1 mm isotropic resolution was obtained using the MPRAGE^28^ (at 3 T) or MP2RAGE^29^ (at 7 T) sequence with the following parameter values. MPRAGE: TR = 2500 ms, TE = 2.15 ms, flip angle = 8°; MP2RAGE: TR = 4300 ms, TE = 2.35 ms, flip angle 1 = 5°, flip angle 2 = 6°. For functional imaging, oblique‐axial slices were placed over the calcarine sulcus following the anatomical reference from MPRAGE (Figure 1B). Based on the anatomical reference, FOV for functional scans were placed to cover primary visual cortex. B_0_ shimming was performed on the imaging volume, using the standard tools provided by the system vendor. Functional data were acquired using an SE‐EPI sequence with and without the PINS module. The sequence parameter values were: TR = 2 s, TE = 75 ms at 3 T and 55 ms at 7 T, number of slices = 8, matrix size = 152 × 152, 1.5 × 1.5 mm^2^ in‐plane resolution, slice thickness = 3 mm with 1.5 mm of slice gaps, GPAPPA = 3, partial Fourier = 6/8, echo spacing = 0.77 ms at 3 T, 0.75 ms at 7 T, volumes = 255. Scans without PINS pulses were implemented by setting the PINS pulse voltage to 0, ensuring that the sequence timing remained identical between experiments with and without PINS pulses.

A visual stimulation was presented, consisting of a flickering checkerboard (8 Hz: 10/30 s ON/OFF). Each functional run lasted 8.5 min having 12 trials. Two runs were acquired for each sequence. Slice timing and motion were corrected using SPM12.^30^ General linear modeling analysis was performed in FSL (https://fsl.fmrib.ox.ac.uk/fsl/) with the default double‐gamma hemodynamic response function.^31^

Voxels with a z‐score above 3.1 were counted as activated voxels. Histograms of the activated voxels from each sequence were plotted together to visualize the effect of inflow on detectability of SE‐BOLD.

## 3 RESULTS

**FIGURE 2:**
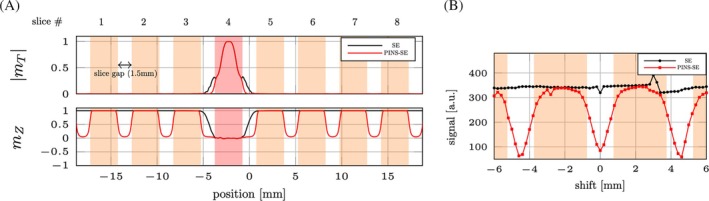
(A) Bloch simulation of the imaging slice profile (top) and steady‐state longitudinal magnetization (bottom) with (red line) and without (black line) power independent of number of slices (PINS)‐saturation pulse. The imaging slice (in this case only slice 4 was simulated) is highlighted in red. (B) Experimental “saturation” profile with (red line) and without (black line) PINS‐saturation module. Imaging slice was moved ±6 mm from isocenter. Shift 0 mm means that center of the imaging slice overlapped with the center of PINS saturation pulse. Both spin‐echo (SE) and PINS‐SE datasets are in same scale.

Figure 2A shows results of the Bloch simulations of the RF pulses. After 9 volume‐TRs, when PINS module was not played (black line), as expected, substantial longitudinal magnetization remains in the slice gaps adjacent to the excitation slice. With the PINS saturation module enabled, the magnetization in the gaps was almost completely saturated (red line).

Figure 2B shows experimentally acquired “saturation” profile. The measured MRI signal was plotted as a function of the excitation pulse shift distance. Specifically, when saturation and excitation slices are overlapped (shift = 0 mm), there was minimum signal left, however, when excitation slices do not spatially overlap with the saturation (shift = ±2.25 mm), maximum signal was observed.

**FIGURE 3:**
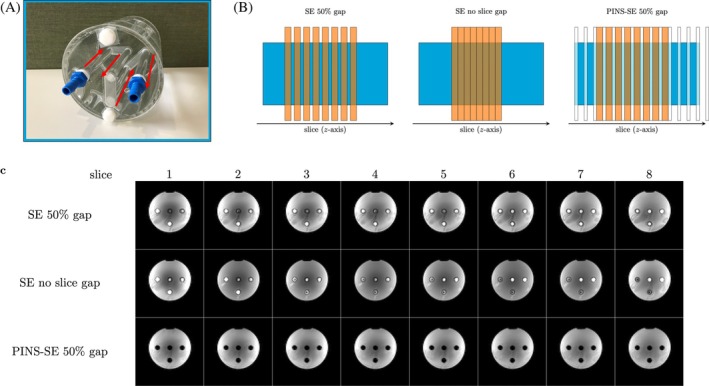
(A) Flow phantom containing 4 tubes with flowing water. The red arrows indicate the water flow direction. (B) Slice placements for spin‐echo (SE) with 50% gap, SE with no slice gap, and power independent of number of slices (PINS)‐SE with 50% gap. The orange and white rectangular shapes represent imaging slices and saturation slices generated by the PINS module, respectively. The light blue square represents the phantom seen from side. (C) Flow phantom images of all 8 slices. When the PINS pulse is not played and there is no slice gap, each of the four tubes show a high signal intensity, indicating an inflow effect. When the PINS saturation module was enabled, the inflow effect was suppressed.

Figure 3C shows results from the flow phantom measurements. As expected, without the PINS saturation module, a bright signal is observed in the tubes with flowing water. Removing the slice gaps does little to suppress inflow effects. Using a 2‐s TR, adjacent slices to the excitation slice were excited 1 and 1.25 s prior because of interleaving slice order [2, 4, 6, 8, 1, 3, 5, 7]. Therefore, there still is ample time for fresh magnetization to flow into the imaging slice. When our PINS saturation module was enabled, inflow effects disappeared from all slices.

**FIGURE 4:**
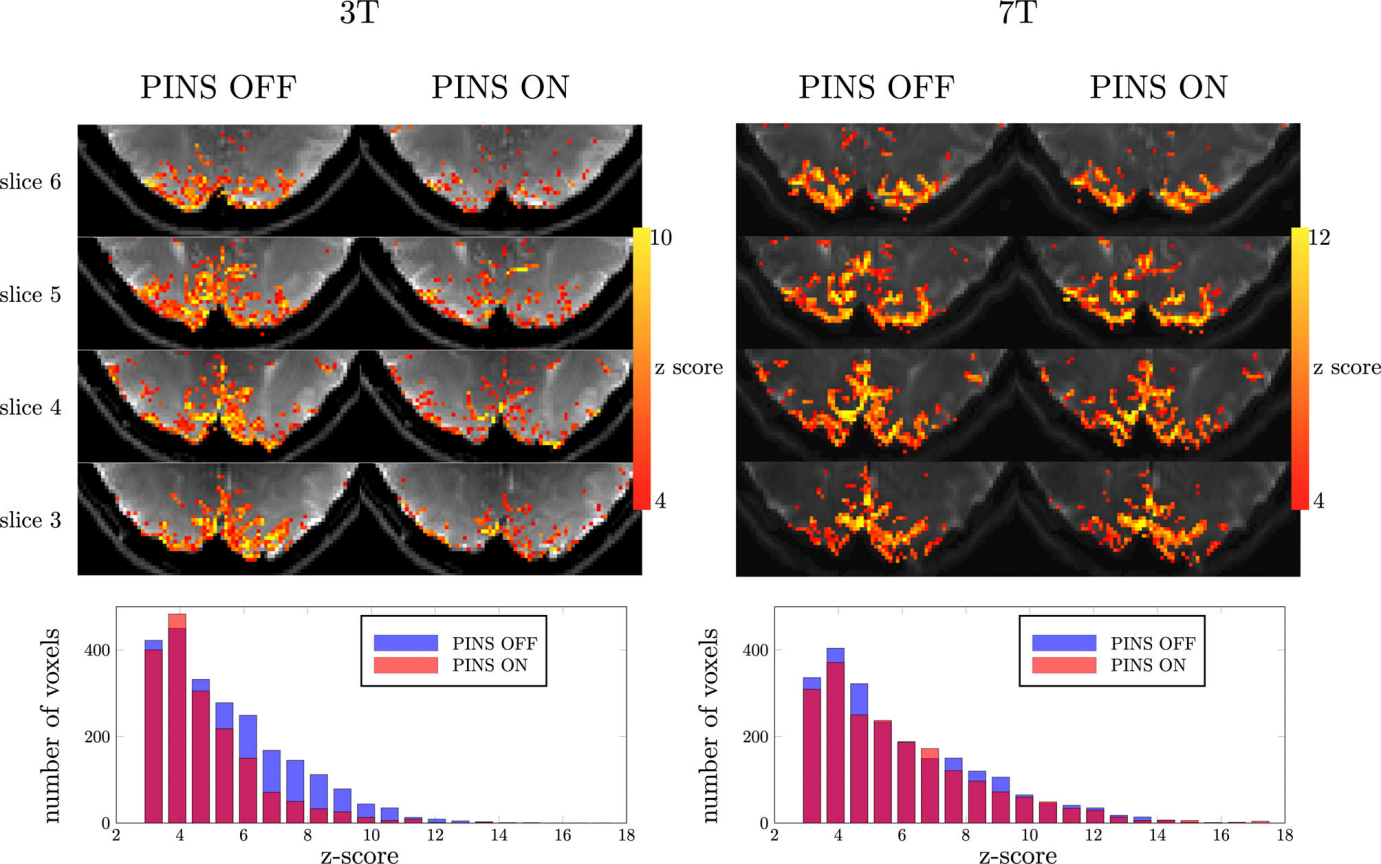
In vivo fMRI results acquired at 3 T and 7 T from same subject. At 3 T, when the power independent of number of slices (PINS) pulse was played, less activation was observed. In contrast, at 7 T, a similar activation pattern and histogram were observed between PINS ON/OFF data.

Figure 4 shows the activation maps and corresponding histograms of the z‐score values from a single subject (results from the other subject can be seen in Figure S1). At 3 T, 25% fewer voxels showed significant activation when PINS saturation module was enabled. However, at 7 T, the difference in activation reduced to 10% (Table 1). The same trend was also observed in subject 2 (see Figure S1). The activation patterns seen with and without PINS saturation were more similar at 7 T.

**TABLE 1:** Number of activated voxels (*z* > 3.1) at 3 T and 7 T for both subjects.

Subject		No. of activated voxels		
		PINS OFF	PINS ON	Ratio
1	3 T	2345	1768	75.4%
	7 T	2237	2029	90.1%
2	3 T	1543	1150	74.5%
	7 T	947	843	89.0%

Abbreviation: PINS, power independent of number of slices.

## 4 DISCUSSION

We have demonstrated that PINS pulses can be used to suppress inflow by saturating the magnetization within slice gaps. Because the PINS saturation module is played before every excitation, a high degree of saturation is possible, even when the volume TR is relatively long. Generally, as TR becomes shorter, the sequence becomes more T_1_ weighted and more sensitive to inflow effects.^7^ However, this PINS module also becomes more efficient as TR decreases. Therefore, for cases in which inflow effects are expected to be strongest, PINS‐saturation can most effectively suppress these effects.

In this work, the PINS pulse was played 8 times per volume, with a volume‐TR of 2 s. With 3‐mm thick slices and a 1.5‐mm slice gap (50%) the flow velocity must exceed 0.6 cm/s to pass through the gap without being saturated (Figure S2). However, the PINS pulse saturates periodically every 3 mm, therefore, the signal from faster moving spins will likely be suppressed as it travels though the stack of many saturation bands, including those outside of the imaging volume.

Conventionally, inflow contributions to fMRI signals have been estimated by comparing signals obtained with different T_1_ weightings.^7^, ^19^ Although such comparisons have helped understand how inflow modulates fMRI signals, the approaches cannot differentiate inflow effects from other T_1_ effects such as CBV changes. In contrast, PINS saturation enabled us to turn on and off the inflow effect by simply setting the transmit voltage to 0 V for PINS module, without changing the imaging module. Therefore, our approach can provide more direct characterization of inflow effects.

In the human brain, blood flow rates in arterioles and venules are 0.1 to 0.6 cm/s.^32^ Therefore, we expected that most of the inflowing intravascular signal was saturated in our study. With this in mind, Figure 4 suggests that inflow effects on SE‐BOLD experiments were more distinct at 3 T than at 7 T. Compared to GRE‐BOLD, SE‐BOLD is weighted more toward the microvasculature. SE‐BOLD acquisitions encompass an extravascular component (because of random motion of water molecules through the local magnetic field variations around the micro vasculature) as well as an intravascular component (because of random motion of water molecules through the local magnetic field variations produces by red blood cells).^33^ Moreover, long EPI readouts can still titrate to some T_2_* weighting into SE‐EPI acquisitions.^34^ Our PINS pulse implementation does not only saturate inflow effects, which contributes a T_1_‐weighted component to nominally T_2_‐weighted BOLD acquisitions, but will also saturate intravascular BOLD signal from any protons flowing into the imaging volume with short delivery times.^35^ At 3 T, the SE‐BOLD signal still contains a significant intravascular component.^13^ Moving to 7 T, the T_2_ of blood in venules and veins decreases more rapidly than the gray matter T_2_, leading to a reduced intravascular component.^13^ Therefore, it was expected that introducing PINS pulses at 3 T would lead to a bigger change than at 7 T. Indeed, at 3 T the number of voxels with z‐score above 3.1 decreased when the PINS saturation module was enabled (˜25%, Table 1). Although we observed a more comparable pattern in the activation maps at 7 T, simply comparing the number of activated voxels (z > 3.1) still suggests a non‐negligible contribution from intravascular BOLD signal (˜10%, Table 1). This observation also agrees with biophysical simulations of the BOLD signal by Uludağ et al.^13^ Note, however, that variations in B_0_ and B_1_ may also contribute to differences observed between 3 T and 7 T.

One of the potential limitations of PINS pulses for inflow saturation is the achievable periodicity and gap size. When a thin imaging slice with small slice gaps is desired (e.g., 1.5 mm), trade‐offs such as the slice profile or total pulse duration must be considered. Theoretically, the PINS saturation module can be used for high periodicity with thin saturation slice thickness, however, it lengthens the pulse duration and increases vulnerability to B_0_ effects. As Figure S3 shows, the PINS pulses used in our experiment start to degrade the imaging slice profiles greater than 15 mm from the center when |dB_0_| is more than approximately 100 Hz. Therefore, caution is advised when optimizing the PINS pulse duration with respect to specific absorption rate. In addition, it should be noted that B_1_ inhomogeneities can result in nonuniform saturation flip angles. Interestingly, the relatively fast repetition rate of the PINS pulses (volume TR/slices) in combination with the long T_1_ of blood^36^ offers some robustness to B_1_ variations (Figure S4). Nevertheless, fast inflowing blood may only see limited number of saturation pulses before entering the image slice. At 7 T, the B_1_ produced by a circularly polarized birdcage can be quite low (˜50% of the target^37^) in the visual cortex. Therefore, despite some inherent B_1_ robustness, B_1_ could factor into the observed differences between 3 T and 7 T experiments. Future work could explore this further using RF‐shimming^38^ or kT‐pins pulses.^39^

In addition, the in vivo the signal was slightly reduced when the PINS pulses were enabled, which was not seen in the phantom scans (Figure S5). The signal change was largest at 7 T in WM, suggestive of a magnetization transfer (MT) effect.^40^ The Fourier transform of PINS pulse contains a wide range of frequencies. Therefore, the PINS pulse likely saturates a larger fraction of protons in macromolecular pool. It may be interesting to explore applications in the context of arterial blood contrast fMRI.^41^

A more obvious limitation of the proposed approach is that it works best when a slice gap is available. When no slice gap is present, it might still be possible to split the acquisition into two measurements or two slice groups with a 100% gap. However, this could lead to increased scan time and misalignment between slice groups to motion. In addition, our PINS pulse only saturates blood signals with relatively short delivery times into the imaging slice. Most likely this will most strongly modulate blood signals in the arterial side of the vascular network. Blood with longer delivery or longer transit times (or moving parallel with the imaging slice) may be modulated less.

To further investigate the contribution of different vascular compartments to the BOLD signal using PINS pulses more detailed experiments are necessary, perhaps with varying imaging resolution and slice prescriptions.^21^ The PINS saturation module can also be implemented into GRE‐BOLD sequences. GRE‐BOLD is more sensitive to larger vessels. Comparing GRE‐ and SE‐BOLD data with and without PINS might provide additional insight into signal contribution from different components of the vascular hierarchy.

## 5 CONCLUSION

Inflow effects from slice gaps were successfully suppressed by PINS‐based saturation modules. To fully remove inflow, it is recommended to use a slice gap in combination with our PINS saturation module, because even multislice sequences without slice gaps can provide ample time for fresh magnetization to flow into the imaging slice. The inflow‐saturated fMRI data highlighted differences in the BOLD signal composition at 3 T and 7 T. The proposed PINS saturation module can be used to further study the contribution of inflow effects in fMRI data.

## CONFLICT OF INTEREST STATEMENT

J.J. is employed by Siemens Healthineers, Australia.

## Supporting information


**Figure S1.** In‐vivo fMRI results acquired at 3 and 7 T from subject 2. At 3 T, when the PINS pulse was played, less activation was observed. In contrast, at 7 T, a similar activation pattern and histogram were observed between PINS ON/OFF data.
**Figure S2.** Relationship between the periodicity of PINS pulses and slice gap required not to experience the PINS pulse.
**Figure S3.** Simulated PINS saturation slice profiles with pulse duration of 7 ms (A) and 10 ms (B). The color scale represents available longitudinal magnetization after the PINS pulse at slice position from iso center with different dB0. The red lines indicate edges of imaging slices.
**Figure S4.** Simulated longitudinal magnetization of blood as a function of number of repetitions at 3 T (A) and 7 T (B). Defining B1 100% corresponds to flip angle of 90°, B1 variations of ± 40% were investigated.
**Figure S5.** Average signal intensity changes in vivo at 3 and 7 T. The table shows relative signal intensity changes in gray matter (GM), white matter (WM), and cerebral spinal fluid (CSF) ROIs.

## Data Availability

The code used to design PINS pulse is available at https://github.com/s‐hodono/PINSpulse_sim.
