# Supplementary material for: Using PINS pulses to saturate inflow effects on fMRI data at 3 and 7 T
Source: Magn Reson Med. 2025 May 20;94(4):1635–43. doi: 10.1002/mrm.30584 (PMC12309871; doi:10.1002/mrm.30584)
Supplement: Supplementary file 1 — Figure S1. In‐vivo fMRI results acquired at 3 and 7 T from subject 2. At 3 T, when the PINS pulse was played, less activation was observed. In contrast, at 7 T, a similar activation pattern and histogram were observed between PINS ON/OFF data. Figure S2. Relationship between the periodicity of PINS pulses and slice gap required not to experience the PINS pulse. Figure S3. Simulated PINS saturation slice profiles with pulse duration of 7 ms (A) and 10 ms (B). The color scale represents available longitudinal magnetization after the PINS pulse at slice position from iso center with different dB0. The red lines indicate edges of imaging slices. Figure S4. Simulated longitudinal magnetization of blood as a function of number of repetitions at 3 T (A) and 7 T (B). Defining B1 100% corresponds to flip angle of 90°, B1 variations of ± 40% were investigated. Figure S5. Average signal intensity changes in vivo at 3 and 7 T. The table shows relative signal intensity changes in gray matter (GM), white matter (WM), and cerebral spinal fluid (CSF) ROIs. [file MRM-94-1635-s001.docx]

Supplementary Materials

Using PINS pulses to saturate inflow effects on fMRI data at 3T and 7T

Shota Hodono^1,2^, Chia-Yin Wu^2,3^, Jin Jin^4^, Jonathan R. Polimeni^5,6^, Martijn A. Cloos^1,2^

1 Donders Centre for Cognitive Neuroimaging, Radboud University, Nijmegen, Netherlands

2 Centre for Advanced Imaging, The University of Queensland, St. Lucia, QLD, Australia

3 Imaging Centre for Excellence, University of Glasgow, Glasgow, United Kingdom

4 Siemens Healthineers Pty Ltd, Brisbane, Queensland, Australia

5 Athinoula A. Martinos Center for Biomedical Imaging, Department of Radiology, Harvard Medical School, Massachusetts General Hospital, Charlestown, MA, 02129, USA

6 Harvard–MIT Program in Health Sciences and Technology, MIT, Cambridge, MA, 02139, USA

**Corresponding Author**

Shota Hodono, Ph.D.

shota.hodono@donders.ru.nl

+31-610-953-049

Donders Centre for Cognitive Neuroimaging, Donders Institute for Brain, Cognition and Behaviour, Radboud University, Kapittelweg 29, 6525 EN Nijmegen


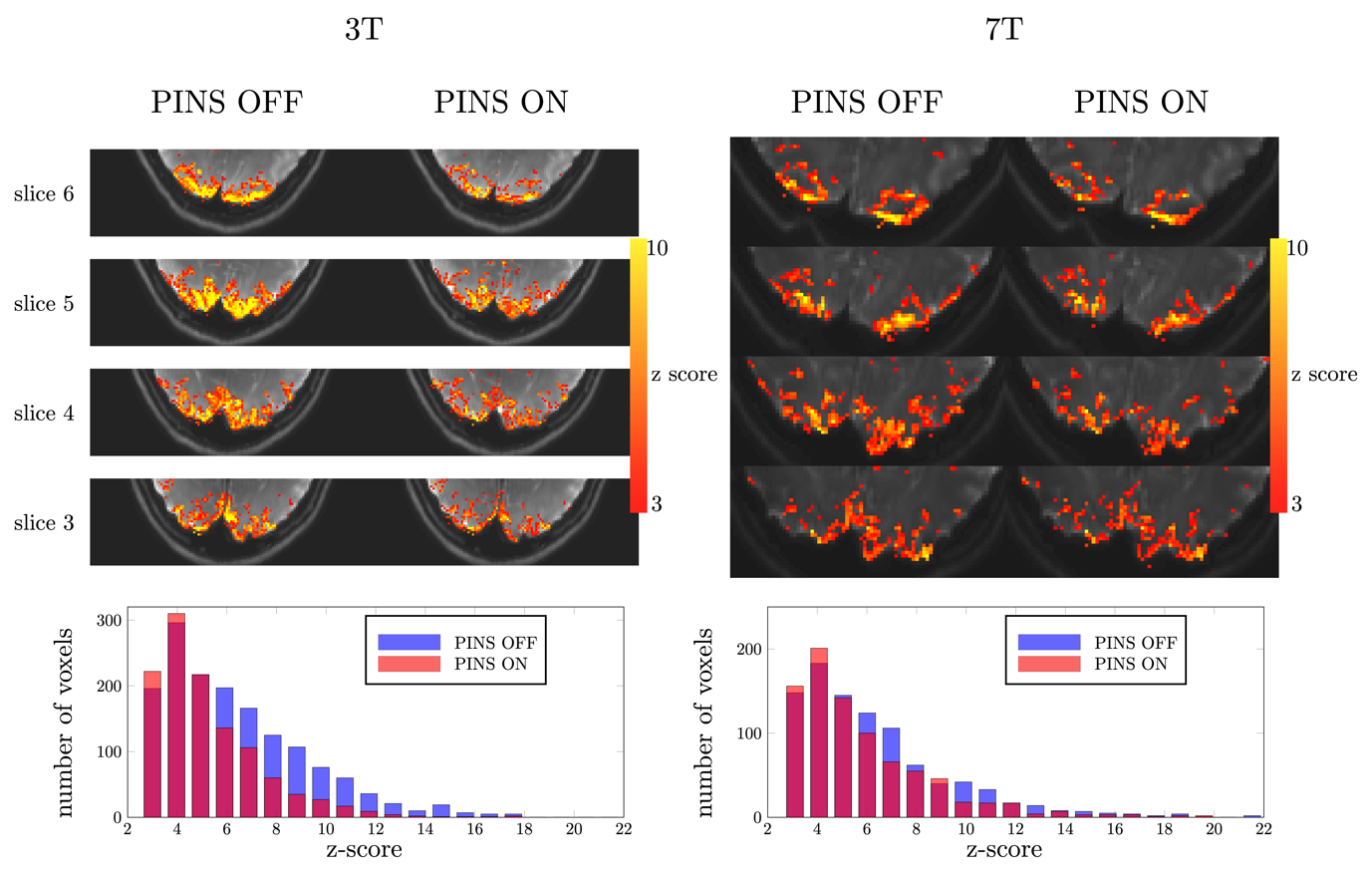


**Supporting Figure S1.** *In-vivo* fMRI results acquired at 3T and 7T from subject 2. At 3T, when the PINS pulse was played, less activation was observed. In contrast, at 7T, a similar activation pattern and histogram were observed between PINS ON/OFF data.


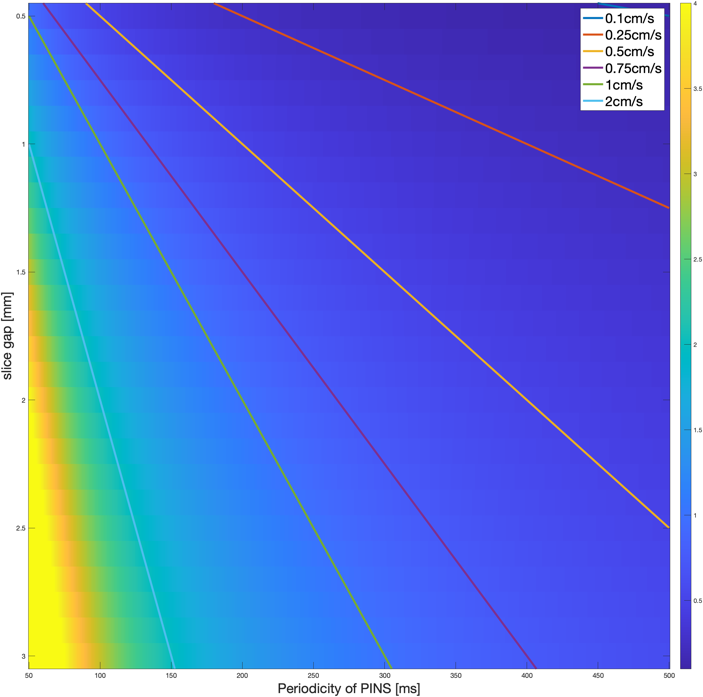


**Supporting Figure S2.** Relationship between the periodicity of PINS pulses and slice gap required not to experience the PINS pulse.


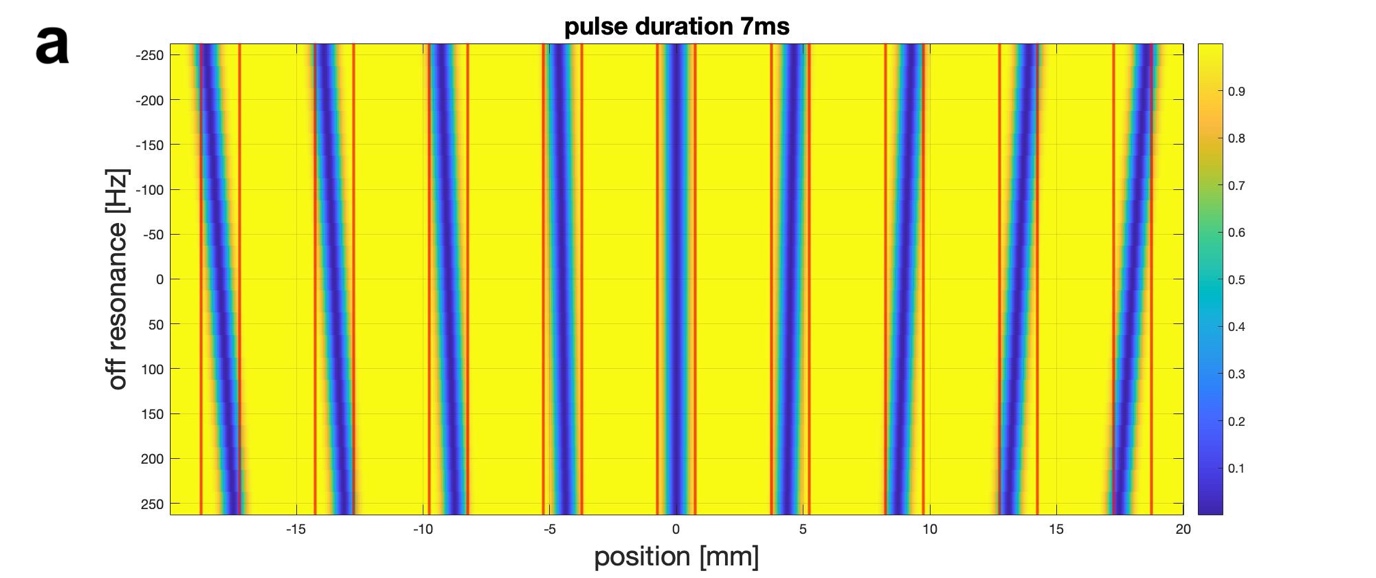


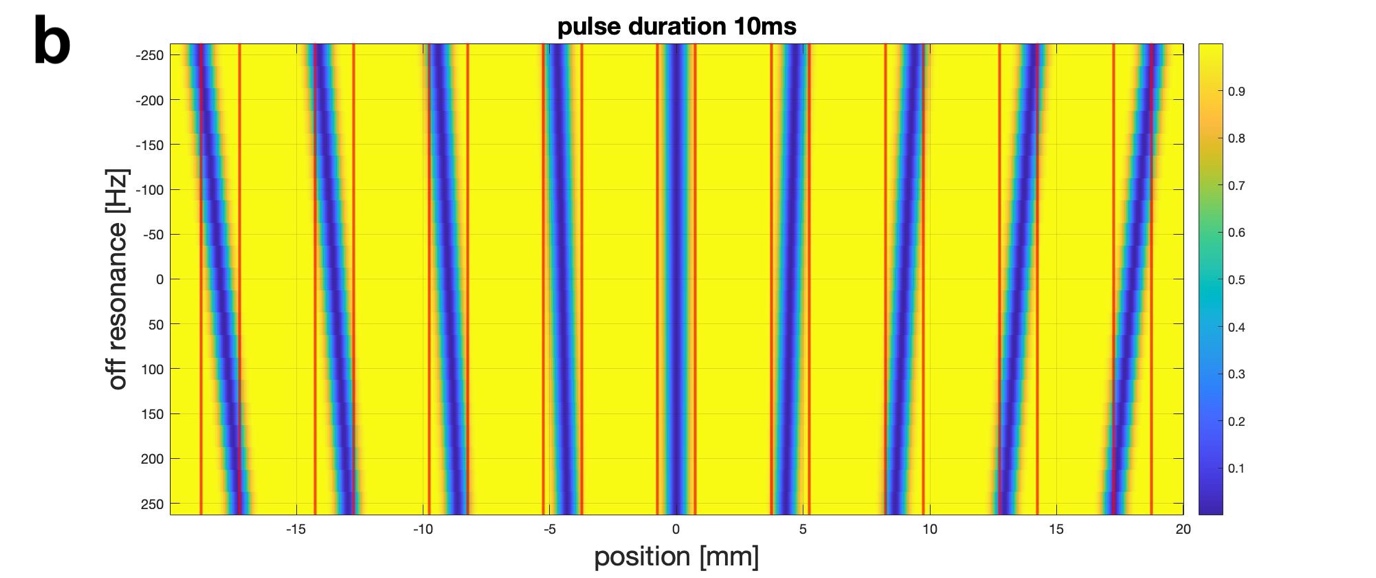


**Supporting Figure S3**. Simulated PINS saturation slice profiles with pulse duration of 7ms (**a**) and 10ms (**b**). The color scale represents available longitudinal magnetization after the PINS pulse at slice position from iso center with different dB0. The red lines indicate edges of imaging slices.


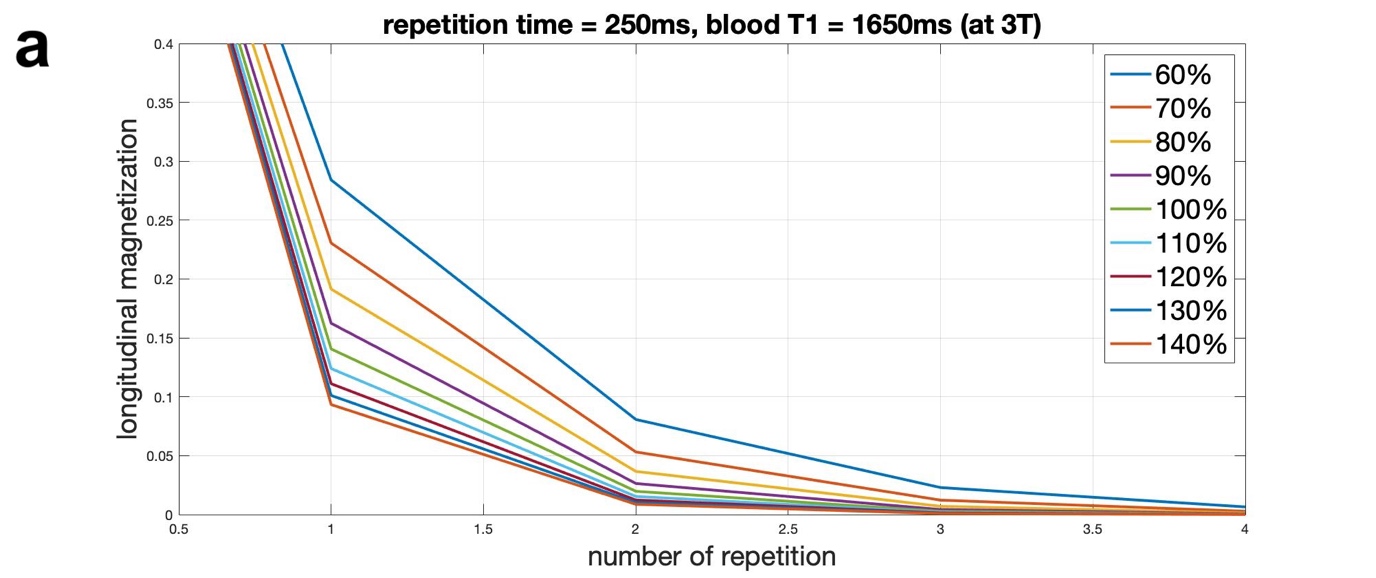


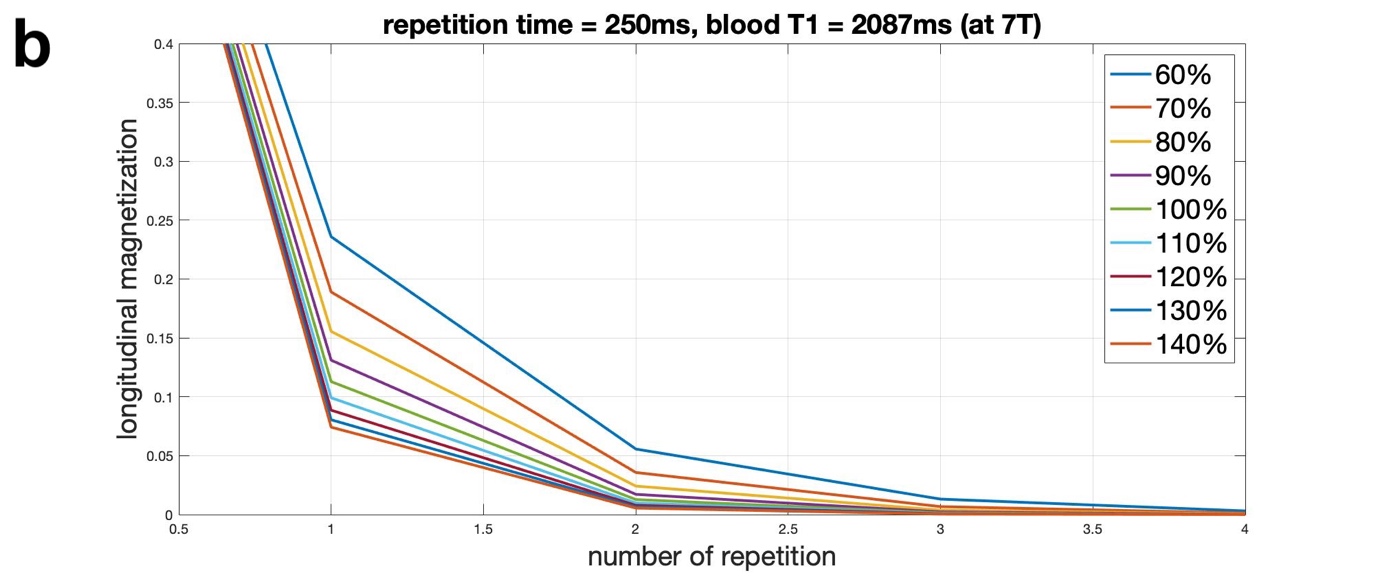


**Supporting Figure S4**. Simulated longitudinal magnetization of blood as a function of number of repetitions at 3T (**a**) and 7T (**b**). Defining B1 100% corresponds to flip angle of 90˚, B1 variations of $\pm$ 40% were investigated.


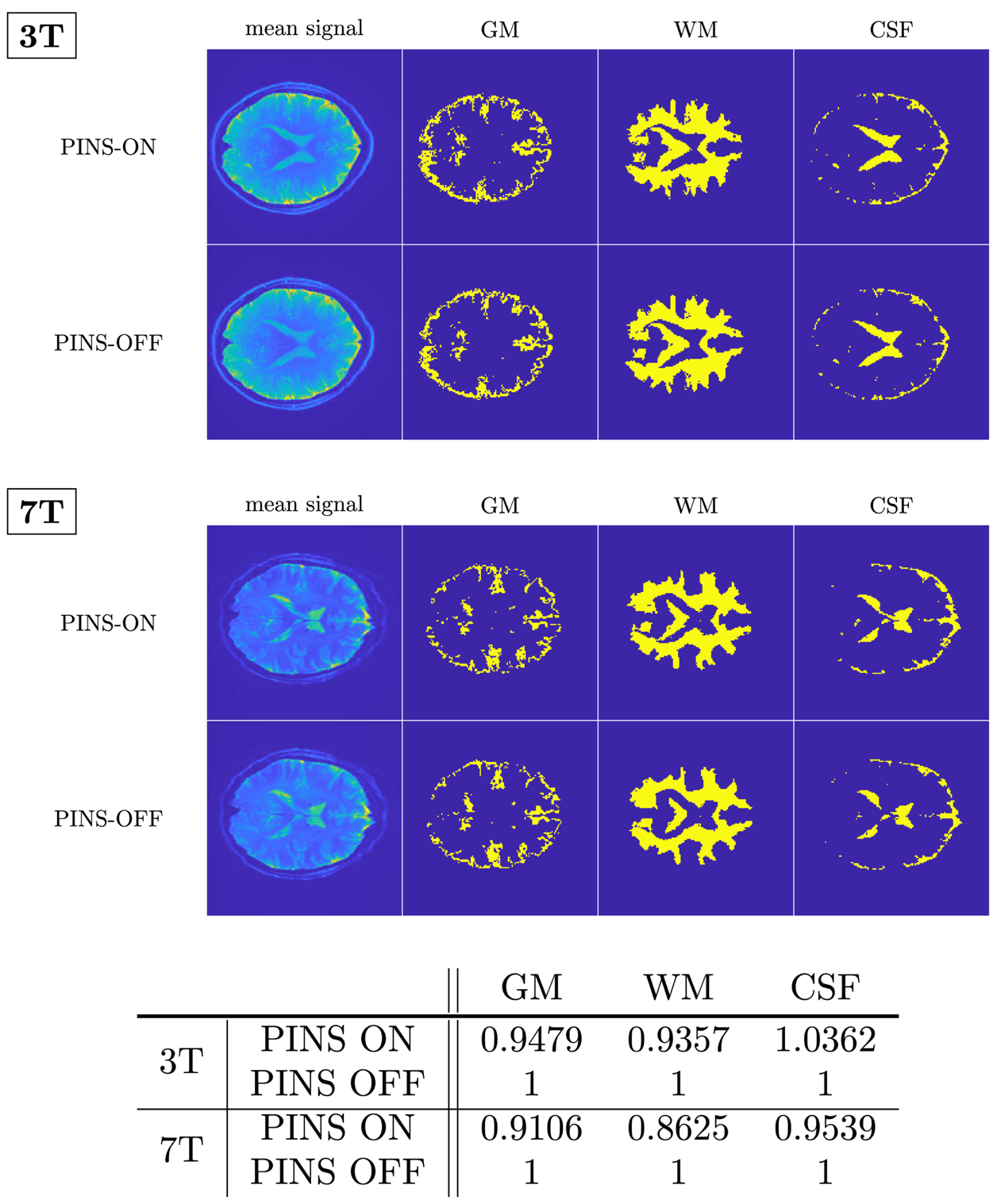


**Supporting Figure S5.** Average signal intensity changes in vivo at 3T and 7T. The table shows relative signal intensity changes in gray matter (GM), white matter (WM), and cerebral spinal fluid (CSF) ROIs
